# Using ecological niche modelling to identify the high-quality ecological areas of *Houttuynia cordata Thunb.* based on secondary metabolites content

**DOI:** 10.3389/fpls.2025.1641634

**Published:** 2025-10-14

**Authors:** Xiaoting Zhang, Jingyi Zhou, Huizhong Xu, Cheng Peng, Fanyun Meng

**Affiliations:** ^1^ Engineering Research Center of Natural Medicine, Ministry of Education, Faculty of Geographical Science, Beijing Normal University, Beijing, China; ^2^ School of Pharmacy, Chengdu University of Tradition Chinese Medicine, Chengdu, China

**Keywords:** *H. cordata*, biomod2, secondary metabolites, spatial analysis, high-quality ecological identifying

## Abstract

**Introduction:**

*Houttuynia cordata Thunb. (H. cordata)*, as a key ingredient in Lianhua Qingwen capsules, plays a critical role in enhancing the antiviral and anti-inflammatory efficacy of the prescription due to its heat-clearing and detoxifying properties, and has contributed significantly to the prevention and control of the COVID-19 pandemic. However, the increasing demand for *H. cordata* - containing Chinese patent medicines and preparations has led to a continuous decline in its wild reserves, which are now insufficient to meet industrial production needs. Current research predominantly focuses on component identification, while the mechanisms underlying the effects of environmental factors on the accumulation of secondary metabolites (SMs) remain poorly understood.

**Methods:**

This study innovatively integrates ecological suitability with a spatial quality assessment system. By employing the Biomod2 ensemble modeling platform to predict the habitat suitability of *H. cordata* and combining path analysis with Kriging interpolation to quantify causal relationships between environmental variables and SMs (including 2-undecanone, quercitrin, and quercetin), we delineated high-quality ecological zones to guide the site selection of artificial cultivation bases.

**Results:**

Key findings include: (1) Ecological suitability zones for *H. cordata* are predominantly concentrated in regions south of the Yangtze River Basin and west of the Hengduan Mountains (e.g., Yunnan-Guizhou Plateau, Sichuan Basin), with annual precipitation (Bio12) and human activities (human footprint, population density) identified as primary drivers. (2) The spatial distribution of three SMs exhibited marked heterogeneity, with no overlapping regions simultaneously achieving high contents of all three metabolites.

**Discussion:**

This spatial divergence underscores the necessity for differentiated cultivation planning based on the pharmacological requirements of target SMs to optimize medicinal value.

## 1 Introduction

*Houttuynia cordata Thunb.* (*H. cordata*), a perennial herb of the Saururaceae family, is widely distributed in humid and shaded environments across East and South Asia, including China, Japan, South Korea, and India. Traditionally valued for its medicinal properties, this plant has been extensively utilized in treating respiratory infections, inflammatory disorders, and gastrointestinal ailments (Chen et al., 2022). In China, its natural habitats span the Yangtze River Basin, Pearl River Basin, and Tibetan Plateau. Modern pharmacological studies have corroborated its therapeutic efficacy, demonstrating antibacterial (Li et al., 2017), antiviral (Yu et al., 2019), immunomodulatory (Cheng et al., 2014), and diuretic activities. Notably, *H. cordata* gained prominence during the COVID-19 pandemic as a key component of Lianhua Qingwen capsules, a specific Chinese medicine formulation renowned for its lung-detoxifying and antiviral effects (Zhao, 2023). The formula synergizes multiple herbs, including *Forsythia suspensa* and *Lonicera japonica*, with *H. cordata* contributing critically to its anti-inflammatory and heat-clearing properties.

The pharmacological potency of *H. cordata* stems from its diverse SMs (SMs), including volatile oils (e.g., methyl heptenone), flavonoids (e.g., quercetin, quercitrin, and hyperoside), and alkaloids. In both traditional Chinese medicine clinical practice and the Chinese herbal pharmaceutical industry, the volatile oil components of *H. cordata* are considered to possess the highest medicinal activity (Pan et al., 2013; Yang et al., 2006). The primary constituent of *H. cordata* volatile oil is methyl-n-nonyl ketone, which is recognized as the main antimicrobial substance, while flavonoids account for 18-35% of the total SMs and demonstrate anti-inflammatory and anti-allergic activities. Studies have shown that flavonoid extracts from *H. cordata* can inhibit the growth of SiHa tumor cells and induce apoptosis (Zhang et al., 2009; Peng et al., 2012; Xue et al., 2013). However, significant geographical variations in SMs composition have been documented, with non-common components distinguishing regional ecotypes (Song et al., 2013). These variations are strongly influenced by ecological factors, underscoring the need to elucidate environment-SM relationships for sustainable resource management.

The superior varieties of medicinal plants form the foundation for producing high-quality natural medicines. Effective conservation and sustainable utilization of medicinal plant resources require precise identification of suitable habitats and an understanding of how environmental factors influence the accumulation of secondary metabolites (SMs) (Teoh, 2015), in order to delineate regions that support both optimal plant growth and high SMs yield. Research indicates that there are significant differences in the main SMs (volatile oil substances and flavonoids) among different sources of *H.cordata*, and there are non-common components that can distinguish the sources or types. The relationship between volatile oil substances and morphological characteristics is closely related, exhibiting latitudinal or longitudinal geographical variations, demonstrating that the ecological environment has a significant impact on the content of its SMs (Song et al., 2013). Therefore, it is necessary to study the potential ecological suitable distribution areas of *H.cordata* herb and high-quality ecological areas based on three SMs.

Species Distribution Models (SDMs) represent a cornerstone methodology in conservation biogeography, enabling the delineation of ecological niches through species-environment relationship modeling (Franklin, 2013) and finding extensive application in critical domains such as endangered species protection (Hu et al., 2020; Qin et al., 2017) and environmental impact assessment. However, the predictive robustness of single-species SDMs exhibits a substantial decline with increasing input data variability (Luo et al., 2017). This limitation can be effectively mitigated through the use of ensemble models. Notably, the biomod2 platform, implemented within the R environment, facilitates ensemble modeling by integrating diverse base models possessing distinct principles, assumptions, and algorithms, thereby enhancing predictive stability and accuracy beyond single-model approaches (Kai-qi et al., 2021; Thuiller, 2003). As one of the most established multi-model frameworks, biomod2 supports robust simulations of species spatial distributions and the identification of key environmental drivers, leading to its widespread scholarly adoption (Guo et al., 2021). For example, Uusitalo et al. (2019) employed biomod2 to model the distributions of *Culex L.* and *Stegomyia Theobald* mosquitoes in the Taita Hills of southeastern Kenya, identifying population density, road distance, and slope as predominant factors influencing Culex, while population density, solar radiation, temperature, and vegetation were more impactful for *Stegomyia*. Similarly, Resquin et al. (2020) utilized biomod2 to investigate the current and potential habitat distributions of *Eucalyptus grandis Hill ex Maiden* and *E. dunnii* Maiden forests in Uruguay, revealing soil surface depth as the primary factor constraining the distribution of both species.

Despite its economic importance in Southwest China as both a medicinal and edible crop (Yang et al., 2013), wild *H. cordata* populations face escalating depletion due to overharvesting and habitat degradation, jeopardizing industrial supply chains (Qi, 2023). While Good Agricultural Practice (GAP) bases aim to standardize cultivation, inconsistencies in medicinal quality—particularly at the Ya’an GAP base—highlight limitations in current practices. While studies have employed species distribution models to investigate the suitable distribution areas of *H. cordata* under current and future climate conditions (Liu et al., 2021), research has not yet explored the effects of environmental factors on its SMs. Establishing cultivation zones that balance ecological suitability with high SMs yield necessitates a dual evaluation framework encompassing both habitat suitability and metabolite optimization.

In this study, we employed the Biomod2 platform to construct an integrated distribution model for *H. cordata*, aiming to identify potential suitable habitats across China and combine SMs analyze the ecological drivers of its spatial quality patterns. The study has three objectives: (1) to identify key environmental determinants of *H.cordata* distribution; (2) to decipher the mechanistic links between abiotic factors and SMs accumulation; (3) to delineate high-quality ecological zones for targeted cultivation. The proposed “ecological suitability–medicinal quality” dual criteria system advances traditional SDM applications, offering a scalable model for GAP base optimization and sustainable utilization of medicinal plants.

## 2 Materials and methods

### 2.1 Study area

The research area is mainly located in the central, southeastern, and southwestern provinces and regions south of the Yangtze River basin in China, including Sichuan Province, Hubei Province, Guizhou Province, Zhejiang Province and others (24°37′N-34°19′N, 97°21′E-123°10’E). This plant mostly grows in tropical and subtropical regions, preferring a shady and humid environment. Its root system is shallow, with few root hairs, and it is highly sensitive to water moisture. Soil humidity should be maintained at 70-80%, and air humidity should be between 60-80%. *H.cordata* prefers loose soil, and sandy loam soil is the most suitable soil type. This plant has low light requirements and is highly shade-tolerant. At the same time, *H.cordata* has a strong temperature adaptability, with its rhizomes able to sprout as long as the temperature is above 12 °C.

### 2.2 Occurrence data, environmental factors and chemical information

#### 2.2.1 Collection of spatial data

The existence data of *H.cordata* were obtained by consulting the Global Biodiversity Information Facility (GBIF, http://www.gbif.org/), the China Virtual Herbarium (CVH, http://www.cvh.org.cn), the National Specimen Information Infrastructure (NSII, www.org.cn), and literature review. Spatial occurrence records spanning a 30-year temporal scope (1990-2020) were rigorously curated for niche modeling implementation. Geospatial precision was ensured by excluding records lacking coordinate data; incomplete occurrences were georeferenced using Google Earth (http://ditu.google.cn/). To minimize anthropogenic bias, only wild populations of *H. cordata* were retained; through literature annotations and online image recognition, cultivated specimens were systematically excluded during the data purification process. Following ecological homogeneity principles where 1 km^2^ grid cells represent functionally equivalent habitats (Zhang et al., 2019), ArcGIS 10.7 (ESRI, Redlands, CA) implemented spatial filtering to eliminate duplicate records within each 1 km×1 km grid, retaining single-point representations per cell. This hierarchical curation protocol yielded a final dataset of 273 spatially independent occurrence points suitable for ecological niche characterization. Using the Biomod2 to generate pseudo-nonexistent data with point positions similar to existing ones for model application, thus enhancing accuracy of the results.

The data points of *H. cordata* samples with SMs come from previous studies. In the literature, there are records of SMs data for multiple periods (Hong et al., 2013; Jiao et al., 2020; Chen and Zheng, 2022; Chen, 2008), and the same processing method as *H.cordata* distribution data is used. In the end, 111 sample points with 2-Undecanone, quercitrin, and quercetin were retained.

**Figure 1:**
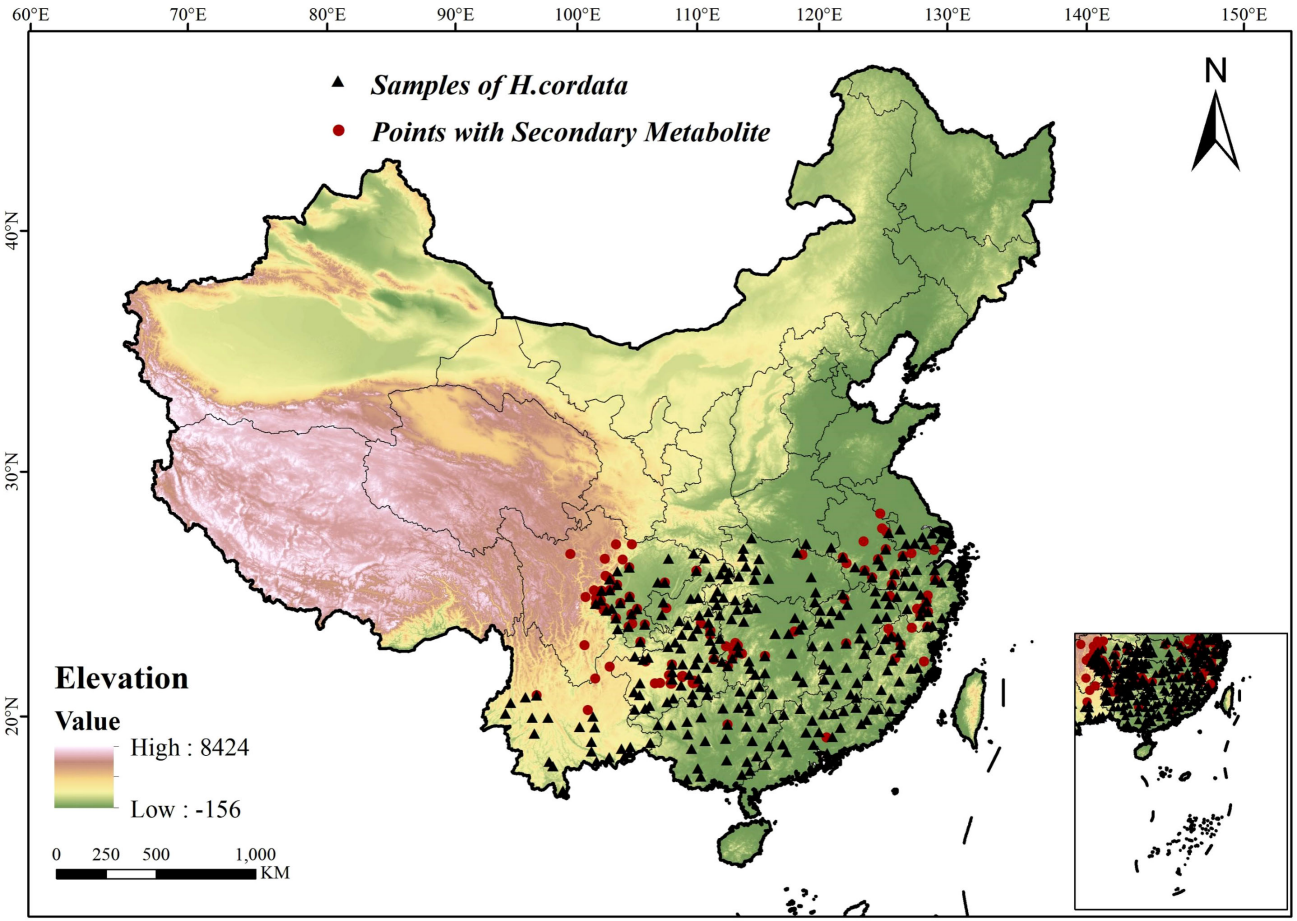
Study area and sampling site distribution.

The spatial distribution diagram of specie sample points and *H.cordata* with SMs is shown in **Figure 1**.

#### 2.2.2 Environmental factors

This study is based on standards such as expert knowledge, ecological knowledge, and exploratory analysis using preliminary statistical models, considering the availability of data and the relevance of biology to select ecological factors relevant to modeling the *H.cordata* (Arenas-Castro et al., 2020; Guo et al., 2018). According to the distribution characteristics of the *H.cordata*, this study initially selected four sets of environmental variables, including climate, soil, topography, and human activities (**Table 1**). Climate Environmental Factors: including 19 derived factors of precipitation and temperature (bio1 - bio19), solar radiation (srad), and vapor pressure (vapr) data, all of which are standard annual average data from 1970 to 2000, downloaded from the Global Climate Database (http://worldclim.org/) (Hijmans et al., 2005). The data downloaded from WorldClim can be directly used for mapping and spatial modeling (Elith et al., 2011). Soil Environmental Factors: Mainly include the spatial distribution data of Chinese soil types (soil), the dataset of Chinese soil characteristics (clay1, clay2, sand1, sand2), and soil quality data (sq1 - sq7) (Wei et al., 2012). The ‘Spatial Distribution Data of Chinese Soil Types’ is sourced from the Resource and Environmental Science Data Center (https://www.resdc.cn/), the ‘Chinese Soil Characteristics Dataset’ is from the National Tibetan Plateau Data Center (http://data.tpdc.ac.cn/zh-hans/), and the ‘Soil Quality Data’ is from the Nanjing Institute of Soil Science, Chinese Academy of Sciences (https://vdb3.soil.csdb.cn/). Terrain Environmental Factors: primarily include elevation (ele), slope (slop), and aspect (asp). Slope and aspect data were generated from elevation data using spatial analysis tools in ArcGIS 10.7 software, while elevation data was sourced from the ENVIREM dataset (Environmental Rasters for Ecological Modeling, https://envirem.github.io/). Human Activity Factors: These encompass the human footprint (hf) (Venter et al., 2016), population density (den) (http://www.ciesin.org/ (accessed 2024.9.1)), and global human-induced changes to the terrestrial system (ter) (https://sedac.ciesin.columbia.edu/data (accessed 2024.9.1)). These variables are derived from socio-economic data and the Application Center (http://sedac.ciesin.columbia.edu).

**Table 1:** Environmental variables used or not used in model.

Variable type	Code (unit)	Description	Variables used in modeling
Climatic variables	bio1(°C)	Annual mean air temperature	
	bio2(°C)	Mean diurnal temperature range (max. temp-min. temp)	✓
	bio3	Isothermality (Bio2/Bio7) × 100	✓
	bio4(°C)	Temperature seasonality	
	bio5(°C)	Max temperature of warmest month	✓
	bio6(°C)	Min temperature of coldest month	
	bio7(°C)	Temperature annual range	✓
	bio8(°C)	Mean temperature of wettest quarter	✓
	bio9(°C)	Mean temperature of driest quarter	
	bio10(°C)	Mean temperature of warmest quarter	
	bio11(°C)	Mean temperature of coldest quarter	
	bio12(mm)	Annual precipitation	✓
	bio13(mm)	Precipitation of wettest month	
	bio14(mm)	Precipitation of driest month	
	bio15(%)	Coefficient of variation of precipitation	✓
	bio16(mm)	Precipitation of wettest quarter	
	bio17(mm)	Precipitation of the driest quarter	✓
	bio18(mm)	Precipitation of warmest quarter	
	bio19(mm)	Precipitation of coldest quarter	
	srad(kJ·m^-2^·d^-1^)	Solar radiation	✓
	vapr(hPa)	Vapor pressure	
Soil variables	soil	Soil type	✓
	clay1	Topsoil Clay Fraction(0 - 30cm)	
	clay2	Subsoil Clay Fraction(30 - 100cm)	✓
	sand1	Topsoil Sand Fraction(0 - 30cm)	
	sand2	Subsoil Sand Fraction(30 - 100cm)	✓
	sq1	Nutrient availability	
	sq2	Nutrient retention capacity	✓
	sq3	Rooting conditions	✓
	sq4	Oxygen availability to roots	✓
	sq5	Excess salts	
	sq6	Toxicity	✓
	sq7	Workability (constraining field management)	
Topographical variables	ele(m)	Elevation above sea level	✓
	slop(%)	Slope	✓
	asp	Aspect	✓
Human activity variables	hf	Human Footprint	✓
	den	Population Density	✓
	ter	Global Human Modification of Terrestrial Systems	✓

All environmental parameters were resampled to a standardized 30-arcsecond spatial resolution (~1 km^2^ ground equivalent; Usher et al., 2004; Venter et al., 2016) to maintain dimensional consistency essential for modeling integrity. Given that multicollinearity among predictors exacerbates model uncertainty, pairwise correlations were quantified using Pearson’s coefficient through the R Corrplot package. This approach systematically retained variables exhibiting absolute correlation values below the established |r| < 0.7 threshold (Dormann et al., 2013) while accounting for dataset non-normality. Variable selection further incorporated *a priori* ecological knowledge of Coriandrum sativum physiology and domain expertise from published literature. This dual-filtering strategy preserved two biologically significant but correlated factors, ultimately yielding 22 optimized environmental predictors for subsequent modeling (**Table 1**).

### 2.3 Methods

#### 2.3.1 Methodology overview

**Figure 2:**
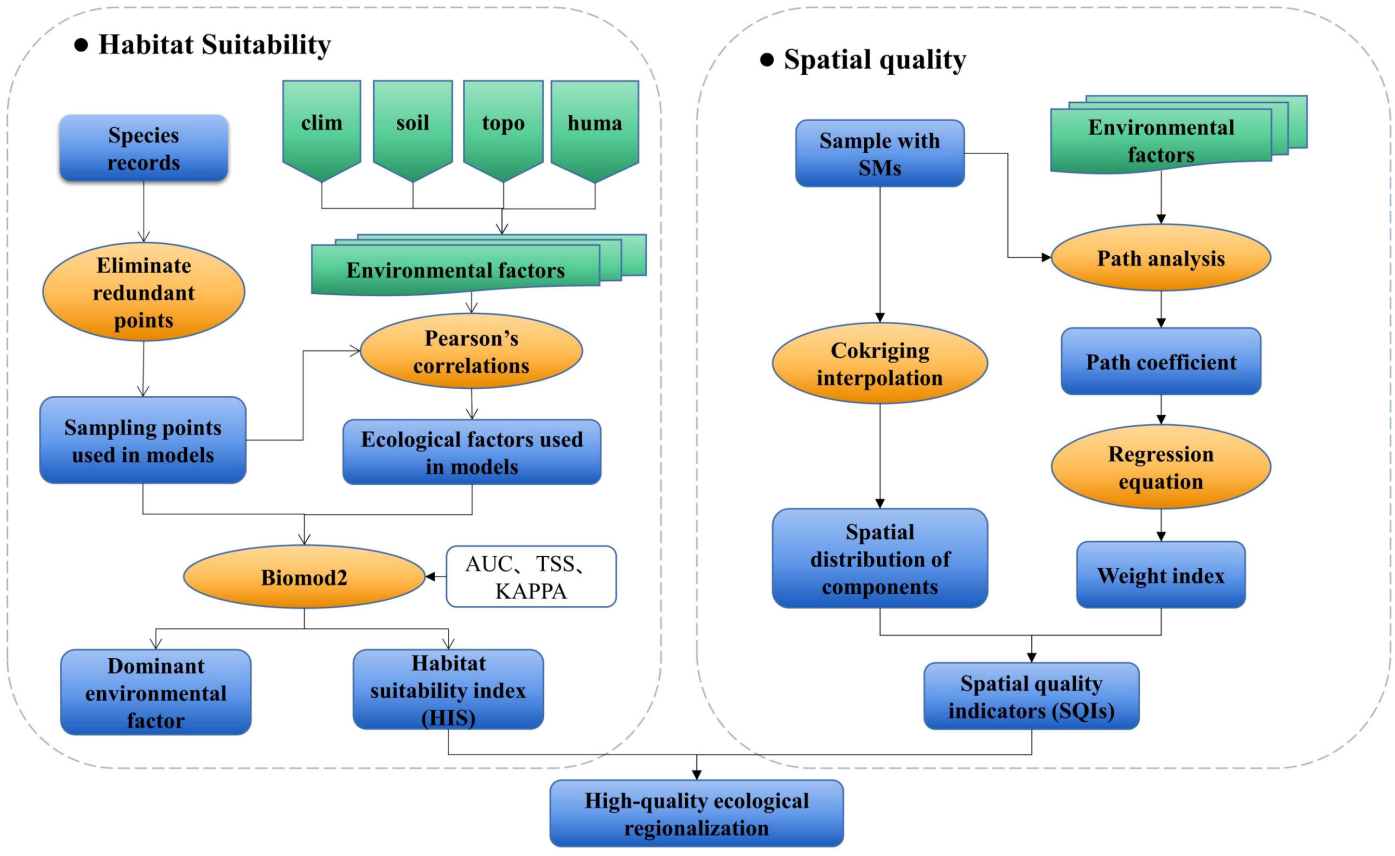
Schematic representation of the methodological approaches.

The spatial distribution of *H. cordata* was modeled using the Biomod2 platform, integrating environmental variables with occurrence data to establish habitat suitability patterns (**Figure 2**). Path analysis - a robust methodology for quantifying direct and indirect causal pathways in multivariate systems (Grapentine, 2000; Suhr, 2008) - was subsequently employed to evaluate environmental drivers of phytochemical quality. This analytical framework generated three integrated indices: (1) Habitat Suitability Index (HSI) derived from Biomod2 outputs; (2) Spatial Quality Indicators (SQIs) developed through path analysis and spatial interpolation of environmental parameters and three key SMs; (3) Ecological Quality Indices (EQIs) constructed by coupling HSI and SQI surfaces (**Figure 2**). Collectively, these metrics establish a multidimensional assessment framework for medicinal plant ecophysiology.

#### 2.3.2 Biomod2 modeling process

Species distribution modeling was implemented using the Biomod2 framework, where ensemble models were constructed through systematic evaluation of individual model performance based on established accuracy metrics. The optimal composite model was subsequently derived from algorithmic selection of top-performing constituent models. This study employed nine distinct single-models within the biomod2 framework for modeling purposes (ANN, CTA, FDA, GBM, GLM, MARS, MAXENT, RF, SRE). To enhance predictive robustness, distribution data were partitioned into training (75%) and validation (25%) subsets using randomized stratification. Three sets of pseudo-absence points were algorithmically (In the “biomod2” package, the BIOMOD_FormatingData function is configured with the argument set to “random”) generated per model run, with 10-fold cross-validation repeated across all configurations. This rigorous validation protocol yielded 300 distinct model realizations (10 iterations × 3 pseudo-absence sets × 10 model replicates).

Model accuracy was assessed using 5-fold cross-validation, with performance quantified through three established metrics: the Area Under the Receiver Operating Characteristic Curve (AUC), the True Skill Statistic (TSS), and Cohen’s Kappa coefficient. The AUC measures discriminative capacity between presence and background points, representing the area under the ROC curve with values ranging from 0.5 (random discrimination) to 1.0 (perfect discrimination); higher values indicate superior model performance. The Kappa coefficient provides a prevalence-adjusted measure of model agreement by incorporating distribution probability, specificity, and sensitivity, yielding values typically between 0 and 1. This metric is particularly valued for its conservatism and robustness in ecological modeling contexts, where values approaching unity signify increasingly reliable predictive accuracy. Its calculation formula is as follows Equations 1–3:


(1) $$KAPPA=\frac{P_{0}-P_{e}}{1-P_{e}}$$

(2) $$P_{0}=P\times Sn+(1-P)\times Sp$$

(3) $$P_{e}=-2(Sn+Sp-1)P(1-P)+P_{0}$$

Among these, *P*, Sn , and Sp represent sensitivity, specificity, and accuracy, respectively; $P_{0}$ stands for observed precision; $P_{e}$ denotes precision occurring by chance (Yunsheng, 2007).

The True Skill Statistic (TSS) represents a derivative metric derived from Cohen’s Kappa coefficient, calculated as the difference between the True Positive Rate (TPR) and False Positive Rate (FPR) with values constrained to the interval [-1,1], where values approaching +1 denote optimal performance. As a prevalence-independent metric, TSS exhibits a significant advantage over the Kappa coefficient by remaining robust to variations in species range size, thereby providing a more reliable measure of discriminatory accuracy across biogeographical contexts. Furthermore, TSS effectively mitigates the confounding influence of species occurrence rates on predictive performance assessments by explicitly distinguishing between true classifications and chance agreement. Equation 4 is the formula for calculating TSS:


(4) $$TSS=\frac{ad-bc}{\left(a+c)(b+d\right)}$$

In Equation 4, a represents the true positive number, b represents the false positive number, c represents the false negative number, and d represents the true negative number.

Define the value ranges corresponding to different levels for three evaluation methods (**Table 2**), providing reference for subsequent model evaluations (Liu et al., 2011).

**Table 2:** Accuracy evaluation criteria of species distribution model.

Evaluation index	Excellent	Fine	General	Low	Fail
KAPPA AUC TSS	0.80-1.00	0.60-0.80	0.40-0.60	0.20-0.40	<0.20
	0.90-1.00	0.80-0.90	0.70-0.80	0.60-0.70	<0.60
	0.80-1.00	0.60-0.80	0.40-0.60	0.20-0.40	<0.20

The final number of single models obtained using Biomod2 depends on the number of successful runs among the 10 models, the predetermined number of pseudo-absences, and the number of repetitions, which is the product of the three. Biomod2 allows custom conditions for model selection among the successful runs, thus selecting single models that meet the criteria to construct a combination model. In this study, single models that performed excellently in the KAPPA, AUC, and TSS evaluation criteria were selected to build the combination model. Using the TSS value, which is more suitable for model evaluation, the weights of each model were calculated. The selected models were then weighted summed to obtain the combination model result.


(5) wi=ri∑i=1i=nri

(6) $$HSI_{j}=\;\sum_{j=1}^{j=n}X_{ij}w_{i}$$

In Equation 5 and 6, $w_{i}$ represents the weight of the i th model, $r_{i}$ represents the TSS value of the i th model, n represents the number of models screened out (meeting KAPPA≥0.80, AUC≥0.90, TSS≥0.80), $HSI_{j}$ represents the ecological suitability index of the j th grid for the combined model, and $X_{ij}$ represents the ecological suitability index of the i th model in the j th grid. Output the model results to obtain the ecological suitability index distribution map of *H.cordata*, showing the degree of adaptation of *H.cordata* in China. Ecological suitability index values, initially ranging from 0 to 1000, were rescaled to the standardized interval [0,1] through division by 1000. Four discrete suitability classes were subsequently established based on threshold values: unsuitable habitat (0–0.2), marginally suitable habitat (0.2–0.5), moderately suitable habitat (0.5–0.7), and highly suitable habitat (>0.7) (Zhang et al., 2024; Zhao, 2024).

#### 2.3.3 Spatial quality modeling

To quantify the influence of environmental variables on SMs accumulation, Spatial Quality Index (SQIs) were derived using ArcGIS 10.7’s Spatial Analyst tools through the integration of Weighted Overlay Path Quality Index (PQIs) and Spatial Interpolation Index (SIIs). Both components were assigned equal weighting (0.5) in the overlay analysis. The specific calculation formula is as follows Equation 7:


(7) SQI=0.5×(POI∩SII)

##### 2.3.3.1 Path quality index, PQIs

To evaluate environmental influences on *H. cordata*’s SMs production beyond traditional niche model contribution rates, path analysis was implemented using environmental variables as independent predictors and three key SMs as dependent response variables. This quantitative approach established direct causal relationships between specific environmental factors and phytochemical compositions through rigorous regression modeling. Correlation analyses among all continuous variables were performed in SPSS software (Zhao et al., 2016) to validate the path model assumptions. The resultant regression equations formally quantify the mathematical relationships between quantified environmental parameters and the measured chemical constituents of *H. cordata*, as shown in Equation 8:


(8) $$C=\beta_{1}x_{1}+\beta_{2}x_{2}+\ldots +\beta_{n}x_{n}+\beta_{0}$$

Among them, the content of C as a SM, $\beta_{i}$ (i≤n), and $\beta_{0}$ are determined using a stepwise regression method, where $x_{n}$ represents the environmental variable (Ferrando and Lorenzo-Seva, 2021).

Building upon prior analytical foundations, path analysis was systematically conducted to examine the three principal SMs. This statistical approach established causality criteria for metabolic relationships by constructing matrix equations through mathematical transformations of bivariate correlation coefficients—both among independent variables and between independent variables and the target metabolites. Ultimately, the method quantified the relative weights of environmental factors governing phytochemical constitutions through standardized path coefficients derived from the structural equation framework. The relevant equations are Equations 9 and 10:


(9) $$\left[\begin{array}{cccc} 1 & r_{x_{1}x_{2}} & \ldots & r_{x_{1}x_{n}}\\ r_{x_{2}x_{1}} & 1 & \ldots & r_{x_{2}x_{n}}\\ \vdots & \vdots & \ddots & \vdots \\ r_{x_{n}x_{1}} & r_{x_{n}x_{2}} & \ldots & 1 \end{array}][\begin{array}{c} \beta_{1}\\ \beta_{2}\\ \vdots \\ \beta_{n} \end{array}]=[\begin{array}{c} r_{x_{1}y}\\ r_{x_{2}y}\\ \vdots \\ r_{x_{n}y} \end{array}\right]$$

(10) $$R_{i}=\beta_{i}^{2}+2\sum i\neq j\beta_{i}r_{x_{i}x_{j}}\beta_{jy}=2\beta_{i}r_{x_{i}x_{j}}-\beta_{i}^{2}$$

$r_{x_{i}x_{j}}$ (i, j ≤ n) represents the simple correlation coefficient between variables, $\beta_{i}$ (i ≤ n) denotes the direct path coefficient of the independent variable $x_{i}\;$ to the dependent variable y , $r_{x_{i}y}$ (i ≤ n) is determined by each independent variable $x_{i}$ relative to the corresponding SMs y .

In addition, we use decision coefficients ${\text{R}}_{\text{i}}$ to assess the extent to which environmental variables lead to the accumulation of SMs. ${\text{r}}_{{\text{x}}_{\text{i}}{\text{x}}_{\text{j}}}{\text{β}}_{\text{jy}}$ represents the Indirect Path Coefficient, and it influences the dependent variable y indirectly through the independent variables ${\text{x}}_{\text{i}}$ associated with ${\text{x}}_{\text{j}}$ . The decision capability $\beta_{\text{i}}^{2}$ varies with the change in values, indicating the path coefficients between environmental variables. To measure the role of key environmental variables in the accumulation of SMs, we calculate the impact weights of environmental variables on SMs based on decision coefficients using weighting coefficients, as shown in specific formula Equation 11:


(11) $$\gamma_{i}=\frac{\left|R_{i}\right|}{\sum_{i=1}^{n}|R_{i}|}$$

This study, based on direct path coefficients, indirect path coefficients, decision coefficients, and environmental variable weights, employs ‘Spatial Analysis’ in ArcGIS 10.7 to calculate the spatial quality distribution of *H. cordata*, namely PQIs (Plant Quality Indices), according to statistical results of SM content.

##### 2.3.3.2 Spatial interpolation index, SIIs

Kriging is an method which can capable of accommodating four variables simultaneously, with one acting as the primary variable and the others as explanatory variables, known as collocated variables. This study employs the Kriging for spatial interpolation. This method combines the spatial auto-correlation of the primary variable with the inter-correlation between the primary and collocated variables, used in unbiased optimal variable estimation, effectively reflecting the impact of multiple environmental factors on the quality of medicinal materials. The expression for Kriging estimated values is as Equation 12:


(12) $${\text{Z}}_{0}=\sum_{\text{i}=1}^{\text{n}}{\text{α}}_{\text{i}}{\text{x}}_{\text{i}}+\sum_{\text{i}=1}^{\text{n}}{\text{β}}_{\text{i}}{\text{y}}_{\text{i}}$$

Among them, ${\text{Z}}_{0}$ is the estimated value of the random variable at position 0; *x_1_,…, x_n_* are the *n* sample data of the initial variable; *y_1_,…, y_m_* are the *m* sample data of the secondary variable; *α_1_,…, α_n_* and *β_1_,…, β_m_* are the Kriging weighting coefficients to be determined.

In ArcGIS 10.7, by utilizing the geostatistical analysis function, the Spatial Interpolation results for *H.cordata* quality based on SMs, SIIs, were obtained.

#### 2.3.4 Ecological quality modeling

To predict the ecological quality zones of *H.cordata* ‘s SMs, it’s necessary to first determine the growth and distribution areas of this species. Then, based on ecological suitability, conduct a spatial quality analysis of the content of its SMs, that is, according to the ecological suitability zoning and spatial quality zoning, to carry out high-quality zoning of *H.cordata* regarding its SM components. Based on the ecological suitability zoning and spatial quality zoning, by fitting ecological suitability and SQIs, calculate the Ecological Quality Index (EQI) of *H.cordata* to construct an ecological quality model regarding its SMs.

The construction of EQIs was initiated by extracting moderately and highly suitable habitats, designated as the HSI, from ecological niche modeling outputs generated via Biomod2. This extraction incorporated all environmental variables previously identified in Section 2.2.2. Subsequently, HSI and SQIs were integrated at a 50% weighting for each index using ArcGIS 10.7 spatial analysis tools to create EQIs (Equation 13) (He et al., 2023). This coupling process, performed at the grid-cell level, simulated spatial distribution patterns of three key SMs. 


(13) $${\text{EQI}}_{\text{i}}=0.5\times (\text{HSI}\cap {\text{SQIs}}_{\text{i}}),\text{i}\leq 3$$

Among them, ${\text{EQI}}_{\text{i}}$ and ${\text{SQIs}}_{\text{i}}$ respectively represent the ecological quality index and spatial quality index of each SM product; HSI is the ecological suitability index determined by the Biomod2 ecological niche model.

## 3 Results

### 3.1 Spatial distribution by ensemble-model of *H. cordata*

**Figure 3:**
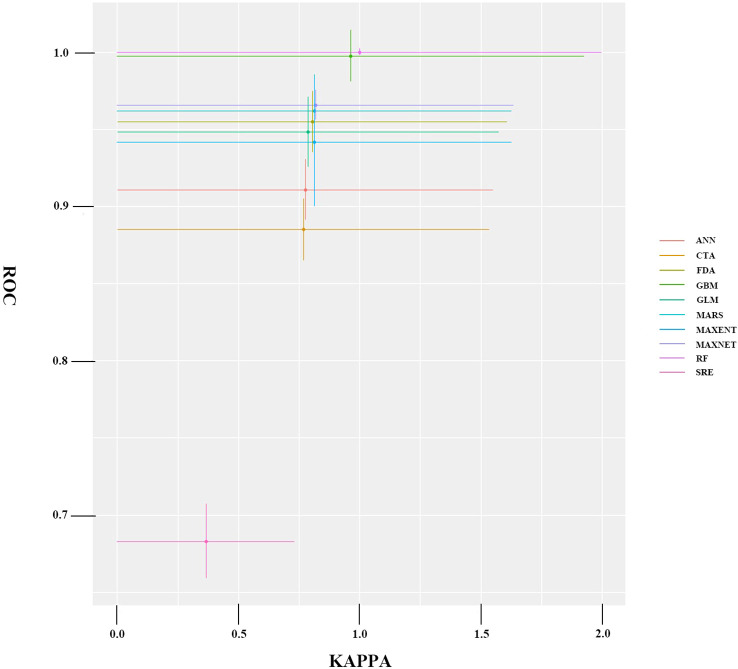
Average accuracy test results for a single model.

Nine distinct species distribution models were successfully implemented within the Biomod2 framework, each generating comprehensive predictive outputs. The comparative performance metrics of these models are presented in **Figure 3**.

The ensemble modeling approach incorporated only the highest-performing individual models from the initial pool of 270 candidate models, applying stringent selection criteria (KAPPA≥0.80, AUC≥0.90, TSS≥0.80) to ensure model robustness. Five models meeting these rigorous standards were integrated into the final composite model, which demonstrated exceptional predictive performance with validation metrics of KAPPA = 0.857 ± 0.04, AUC = 0.975 ± 0.06, and TSS = 0.861 ± 0.03. The resulting suitability predictions exhibited biologically meaningful spatial patterns across all classification levels, showing smooth ecotonal transitions between habitat quality categories.

**Figure 4:**
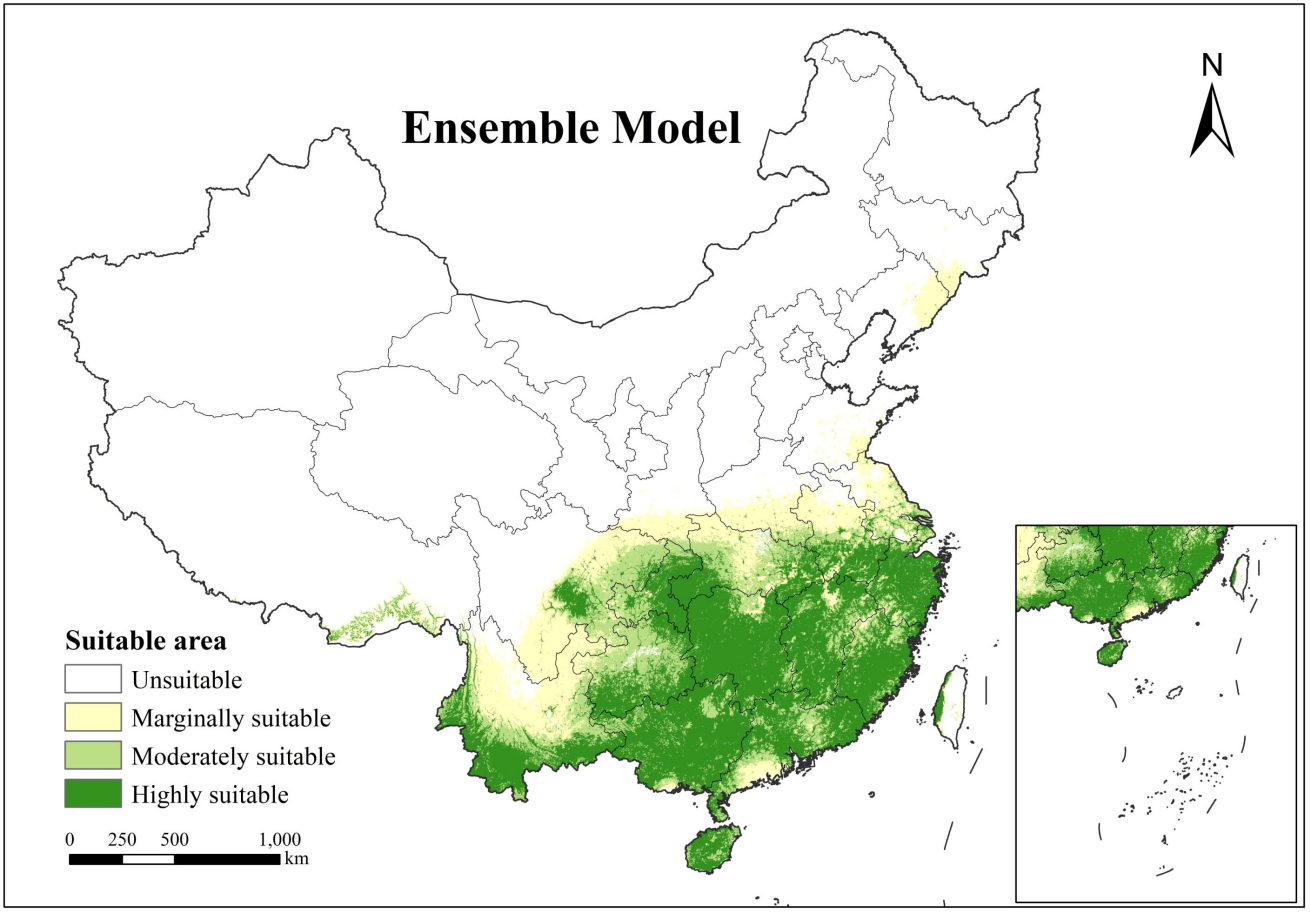
Spatial distribution of *H. cordata* ecological suitability simulated by the ensemble model.

The composite modeling outputs (**Figure 4**) exhibit coherent spatial patterning across all habitat suitability classes, demonstrating structural coherency with smooth transitions between classification tiers. This integrated model achieves high spatial contiguity while maintaining distinct hierarchical differentiation, effectively delineating the ecological suitability distribution of *H. cordata* across China. Geospatial analysis reveals primary suitable habitats concentrated predominantly south of the Yangtze River Basin and west of the Hengduan Mountains, encompassing the Yunnan-Guizhou Plateau, Sichuan Basin, and Southeastern Hills. Core distribution zones (moderate to high suitability) cluster predominantly in eastern Sichuan’s Ya’an region, the Chongqing-Hubei border interface, southern Yunnan, northwestern Taiwan, and throughout Hainan, Guangxi, Hunan, Zhejiang, and Fujian provinces. Marginal suitability areas form transitional buffers along peripheries of medium-high suitability zones, with isolated occurrences observed in southern Tibetan river valleys and limited sectors of Shandong and Liaoning provinces. According to the composite model results, the highly suitable area for *H.cordata* in China covers an area of 1.09×10^6^km^2^, the moderately suitable area covers an area of 5.4×10^5^km^2^, and the marginally suitable area covers an area of 5.5×10^5^km^2^. The area of the highly suitable region is roughly equal to the combined area of the moderately and marginally suitable regions.

### 3.2 Spatial quality indicator for *H. cordata*

#### 3.2.1 Result of path analysis

**Table 3** displays the correlation between environmental variables and three SMs (2-Undecanone, quercitrin and quercetin) as revealed through correlation and regression analyses.

**Table 3:** Path coefficients of environmental variables and three SMs.

(a) 2-undecanone										
Environmental variables		Indirect path coefficient						Accumulating contribution	Decision-making	Index weight
		Bio7	Bio12	Slope	Bio8	Sq4	Sq2			
Bio7		/	0.037	0.067	-0.004	-0.07	0.024	-0.610	0.308	0.115
Bio12		0.049	/	0.106	-0.028	0.034	0.050	-0.046	0.395	0.148
Slope		0.104	0.124	/	0.131	0.068	-0.04	-0.400	0.436	0.163
Bio8		-0.020	-0.060	0.256	/	-0.08	0.016	-0.206	0.486	0.182
Sq4		-0.160	0.060	0.103	-0.064	/	0.069	-0.270	0.514	0.192
Sq2		-0.090	-0.140	0.106	-0.020	-0.110	/	0.172	0.533	0.199
(b) Quercitrin										
Environmental variables	Indirect path coefficient							Accumulating contribution	Decision-making	Index weight
	Bio7		Den				Sand2			
Bio7	\		0.046				-0.018	0.174	0.344	0.367
Den	-0.060		\				0.074	0.356	0.311	0.332
Sand2	-0.038		-0.010				\	-0.351	0.282	0.301
(c) Quercetin										
Environmental variables	Indirect path coefficient							Accumulating contribution	Decision-making	Index weight
	Bio7		Sand2							
Bio7	\		0.053					0.492	0.246	0.413
Sand2	0.049		\					0.322	0.35	0.587

For 2-undecanone synthesis, thermal amplitude (Bio7), wet-season temperatures (Bio8), and annual precipitation (Bio12) emerged as critical climatic regulators, paralleling their established roles in determining the species’ ecological suitability. Edaphic factors—including slope-mediated drainage patterns, nutrient retention capacity (Sq2), and root-zone oxygen availability (Sq4) — were equally pivotal, aligning with recent soil-plant interaction studies (Qi, 2023). The flavonoid derivatives quercitrin and quercetin exhibited contrasting environmental associations, demonstrating strongest correlations with thermal variability (Bio7), subsurface sand content (Sand2), and population density (Den). Notably, the persistent influence of thermal amplitude (Bio7) across multiple metabolite classes suggests fundamental physiological constraints on biochemical pathways, potentially mediated through temperature-sensitive enzymatic processes. Based on regression coefficients, a regression equation between SMs and environmental variables can be established, and it’s found that the equation can explain their mathematical correlation, as shown in Equations 14–16:


(14) $${\text{y}}_{1}=1.573-0.61{\text{x}}_{1}-0.206{\text{x}}_{2}-0.466{\text{x}}_{3}-0.4{\text{x}}_{4}+0.172{\text{x}}_{5}-0.27{\text{x}}_{6}$$

(15) $${\text{y}}_{2}=0.471+0.174{\text{x}}_{1}+0.356{\text{x}}_{7}-0.351{\text{x}}_{8}$$

(16) $${\text{y}}_{3}=-2.54+0.492{\text{x}}_{1}+0.322{\text{x}}_{8}$$

Among them, ${\text{y}}_{1}$ , ${\text{y}}_{2}$ and ${\text{y}}_{3}$ respectively represent 2-Undecanone, quercitrin and quercetin. ${\text{x}}_{1}$ - ${\text{x}}_{8}$ correspond to Bio7, Bio8, Bio12, Slope, Sq2, Sq4, Den, and Sand2.

The synthesis of 2-undecanone showed predominant dependence on Sq4, Sq2, and Bio8, with Bio12 and Slope gradient exhibiting complementary positive associations. Notably, Bio7 demonstrated inhibitory effects on this volatile compound’s accumulation. Contrastingly, flavonoid production revealed differential controls: quercitrin concentrations correlated positively with Sand2 and Den, while both quercitrin and quercetin showed temperature-mediated regulation through Bio7 fluctuations. The persistent significance of Bio7 across all three SMs underscores its fundamental role in modulating biosynthetic pathways, potentially through temperature-sensitive enzymatic kinetics or stress-induced metabolic responses.

Based on the contributions of different variables to three types of SMs, the weight indices of environmental variables for each SM were also calculated through path analysis. Based on the obtained weights, we used ArcGIS 10.7 to locate the PQIs of *H.cordata*.

#### 3.2.2 Result of spatial quality distribution

**Figure 5:**
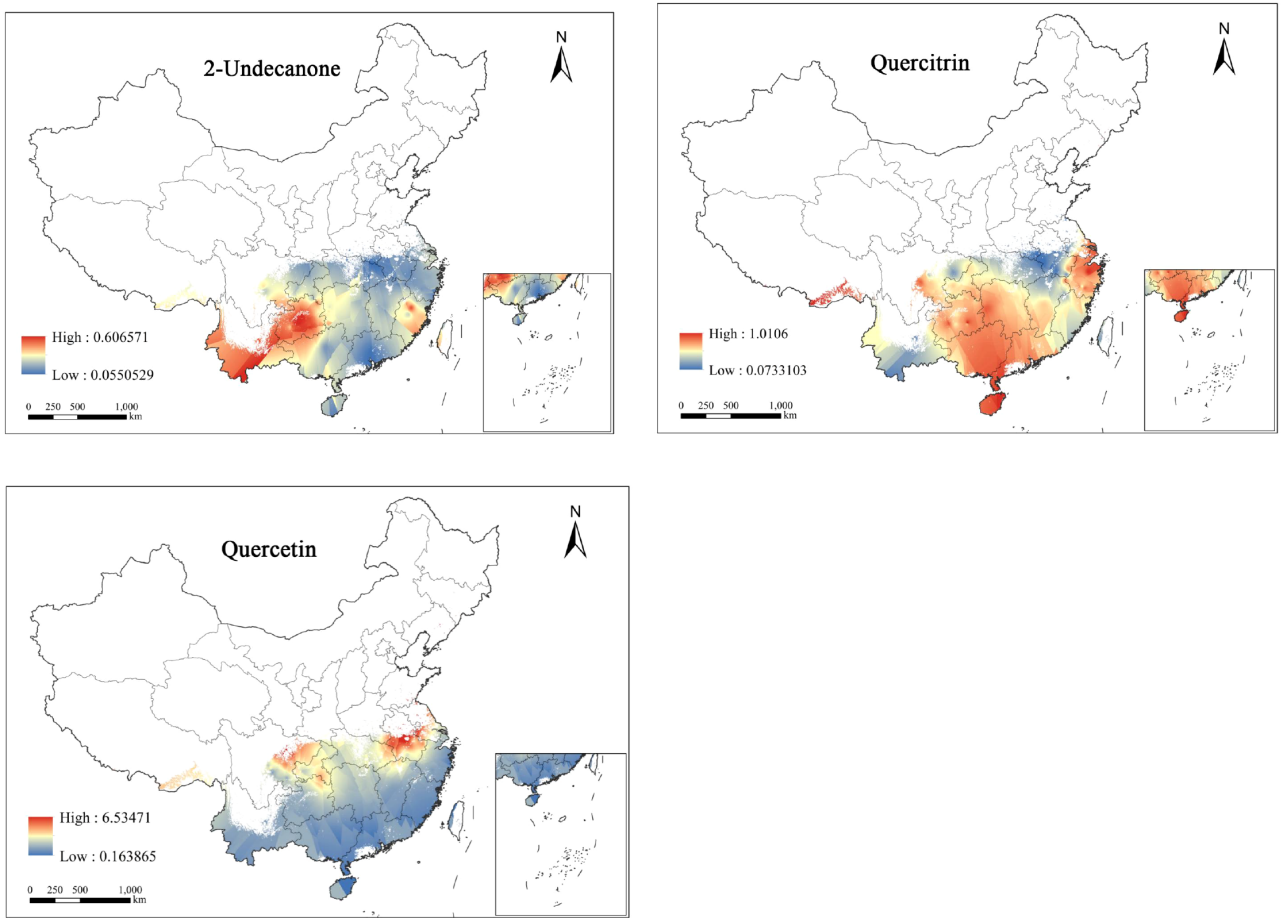
Spatial mass distribution map of *H.cordata* based on SMs.

By applying the CC method to interpolate the spatial quality of three SMs of *H.cordata*, the resulting SIIs were overlaid with the PQIs obtained from pathway analysis to generate a spatial quality distribution map of the three SMs (**Figure 5**).

Predicted concentrations of three SMs exhibit spatial variability. The concentration range of 2-Undecanone is 0.05-0.60mg/g, with its high-concentration areas primarily located in central Guizhou and southern Yunnan, and parts of Fujian also relatively high. The concentration range of quercitrin is 0.07-10.1mg/g, with its high-concentration areas more widely distributed, extending from Sichuan, Guizhou, Guangxi, Hunan, and Hainan to most of Zhejiang, marking the high-concentration zones of quercitrin. The concentration range of quercetin is 0.16-6.51mg/g, yet its high-concentration areas cover the smallest area, only found in eastern Sichuan, Chongqing, and the southwestern part of Hubei, at the peripheral regions.

Considering the distribution of three types of SM contents, areas with high concentrations of a single SM are relatively concentrated, with no clear pattern of decrease from high to low, and almost no region shows high contents of all three types of SMs simultaneously. Therefore, in actual cultivation, different priority planting areas can be determined based on the varying demands for SMs, to maximize their value.

### 3.3 High-quality ecological regionalization for *H.cordata*

**Figure 6:**
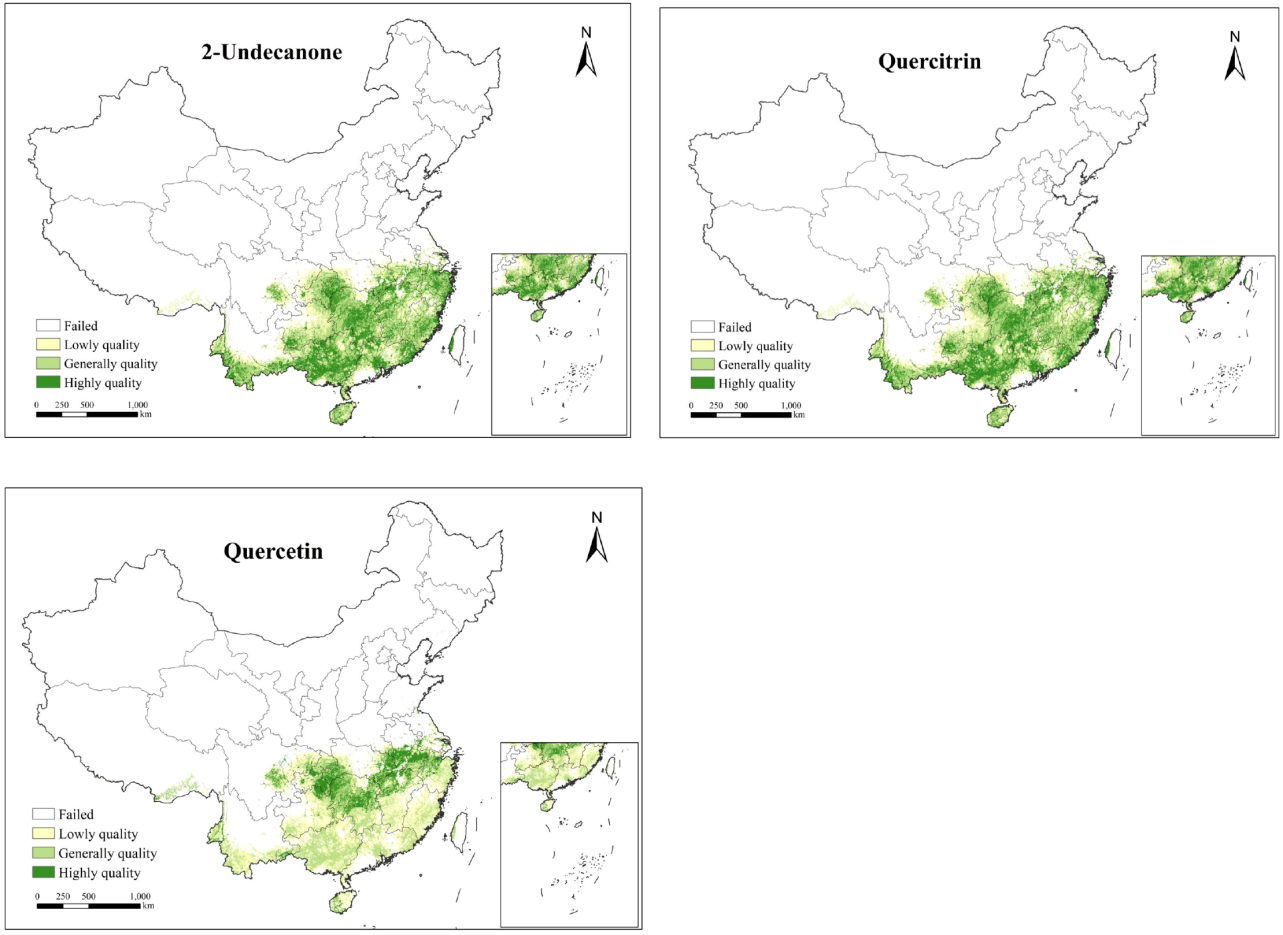
Ecological quality distribution map of *H.cordata* based on SMs.

This study uses HSI as a constraint region, limiting the predicted range of 2-Undecanone, quercitrin and quercetin content in *H.cordata* to areas with higher growth suitability. Based on EQIs results, this study classifies the ecological zones for high-quality SMs of *H.cordata* into four levels: unsuitable areas, low quality areas, generally quality areas, and highly quality areas. The total area of generally and highly quality zones for 2-Undecanone is approximately 9.2×10^5^km^2^, for quercitrin approximately 1.09×10^6^km^2^, and for quercetin approximately 4.0×10^5^km^2^ (refer to **Table 4** and **Figure 6**).

**Table 4:** Ecological quality area statistics of main SMs of *H.cordata*.

Ecological quality	2-undecanone(×10^5^km^2^)	Quercitrin(×10^5^km^2^)	Quercetin(×10^5^km^2^)
Highly quality	4.58	4.57	0.98
Generally quality	4.61	6.35	3.03
Lowly quality	5.26	3.52	8.25
Unsuitable	2.32	2.32	3.50
Generally and highly	9.19	10.9	4.02

The spatial distribution of high-quality ecological zones for SMs in *H. cordata* exhibits distinct geographical patterns with significant regional specificity. High-quality zones for 2-undecanone are predominantly clustered along the Chongqing-Hubei border, southwestern Guizhou, and northwestern Guangxi, reflecting optimal edaphic and microclimatic conditions for volatile oil biosynthesis. Quercitrin-rich zones demonstrate partial spatial overlap with 2-undecanone hotspots but exhibit pronounced longitudinal gradients, displaying superior quality metrics in western regions—particularly southwestern Hubei—with gradual declines toward eastern areas. Conversely, quercetin optimization zones occupy the smallest spatial footprint, primarily localized along the northern peripheries of the species’ ecological suitability range, including southwestern Hubei and adjacent transitional ecotones, exhibiting latitudinal quality stratification with enhanced northern productivity. These findings align with Yiling Pharmaceutical’s recent establishment of a *H. cordata* cultivation base in the Chongqing-Hubei border region, validating the methodological rigor and translational value of this ecological quality assessment framework.

## 4 Discussion

The reliability of ecological niche models fundamentally depends on the precision of input datasets, particularly the comprehensiveness of environmental variables and georeferenced occurrence records, yet data acquisition for critical ecological parameters remains a persistent challenge in species distribution modeling (Sillero and Barbosa, 2020; Wisz et al., 2008). Compounding these complexities, interspecific variations in survival strategies and niche specialization introduce substantial uncertainties in defining species-environment relationships (Williams et al., 2012). To address this fundamental challenge, the present study simulated *H. cordata*’s ecological niche and spatial distribution through integrative analysis of environmental variables and occurrence data. This modeling framework based on comprehensive distribution point sampling. By isolating biogeographic drivers from confounding anthropogenic factors, the approach derives a baseline understanding of the species’ fundamental niche dimensions. By integrating environmental predictors with verified occurrence records, we developed a mechanistic understanding of niche determinants while systematically evaluating variable contributions through dual ecological and phytochemical lenses. The EQIs not only confirmed environmental regulation of SMs biosynthesis but also revealed differential variable dominance across metabolic pathways, demonstrating how microhabitat conditions interactively govern phytochemical profiles.

### 4.1 Effects of environmental factors on ecological suitability of *H. cordata*

The environmental drivers analysis revealed (**Table 5**) precipitation as the dominant factor governing *H. cordata* distribution, with Bio12 and Bio17 consistently ranking as top predictors across models. This hydrological dominance aligns with the species’ core distribution in China’s subtropical monsoon zone, where regions exceeding 800 mm annual rainfall - including peripheral areas like southern Tibetan valleys and Liaoning’s coastal zone - provide essential moisture conditions for its perennial growth cycle. The species’ phenological strategy, characterized by extended vegetative periods (May-October flowering/fruiting) and persistent rhizome systems, demonstrates evolutionary adaptation to stable precipitation regimes that maintain soil moisture through seasonal variations.

**Table 5:** Environmental variables and contribution of the single models.

Models	Environmental variables and contribution (%)					Accumulating contribution (%)
ANN	Bio12	Bio17	Elev	Srad	Den	90.7
	57.2	12.2	8.7	6.3	6.3	
CTA	Bio12	Bio17	Ter	Elev	Hf	84
	69.2	8.7	2.3	2.1	1.7	
FDA	Bio12	Bio17	Bio2	Sq2	Ter	93.6
	39.4	26.4	10.2	9.5	8.1	
GBM	Bio12	Bio17	Bio2	Ter	Hf	42.7
	23.2	10.1	5.3	2.7	1.4	
GLM	Bio12	Bio2	Bio7	Bio17	Bio15	92.7
	47.1	22.3	16.5	4.7	2.1	
MARS	Bio12	Bio17	Bio2	Ter	Hf	74.9
	25.1	23.9	9.8	9.1	7.0	
MaxEnt	Bio12	Bio17	Hf	Elev	Den	90
	60.4	15.0	7.3	3.7	3.6	
RF	Bio12	Bio17	Bio7	Bio2	Den	68.3
	28.2	24.6	8.9	5.3	1.3	
SRE	Bio12	Bio17	Bio7	Bio2	Den	93.7
	30.5	30.2	28.3	2.4	2.3	

Topographic influences (Elev) operated indirectly through microclimate modulation, particularly via elevation-mediated temperature-precipitation gradients, rather than exerting direct physiological constraints. Contrary to expectations, soil parameters showed minimal predictive power, attributable to *H. cordata*’s broad edaphic adaptability - thriving optimally in moist, humus-rich sandy loams while maintaining viability in clay-dominated substrates (wu et al., 2008). Furthermore, human footprint (Hf) and population density (Den) frequently ranked among the top five important variables, demonstrating the species’ tolerance to moderate anthropogenic disturbance alongside its vulnerability to habitat fragmentation in rapidly urbanizing landscapes. These findings collectively underscore the primacy of macroclimatic moisture availability over localized edaphic conditions in shaping the species’ ecological niche, and anthropogenic pressures introduce partial modifiers to distribution patterns in human-modified ecosystems.

### 4.2 The key role of environmental factors in SMs

Path analysis revealed distinct environmental drivers for specific SMs in *H. cordata*, with Slope, Sq2 and Sq4 exerting significant control over 2-Undecanone accumulation. Conversely, Bio7 and Sand2 emerged as critical determinants of quercitrin and quercetin concentrations. This metabolite-specific environmental sensitivity underscores the complex interplay between abiotic factors and phytochemical biosynthesis, where temperature regimes, hydrological conditions, and edaphic properties collectively modulate metabolic pathways through both direct physiological effects and indirect microhabitat modifications.

The ecological significance of these SMs extends beyond plant adaptation, as they represent valuable bioactive compounds with documented pharmaceutical applications (Zhao et al., 2005). Plants strategically modulate SM production as an evolutionary response to environmental stressors (Bennett and Wallsgrove, 1994), with *H. cordata*’s flavonoid profile particularly sensitive to geo-climatic variations. The species’ preferential accumulation of quercitrin over other flavonoids (Apple et al., 2000) suggests targeted biosynthetic investment, potentially linked to its enhanced antioxidant capacity and therapeutic efficacy. This spatial heterogeneity in flavonoid composition, correlated with morphological adaptations across populations (Wu et al., 2008), necessitates geolocation-specific cultivation strategies to optimize medicinal quality.

Notably, 2-Undecanone biosynthesis demonstrates strong edaphic dependence, reflecting *H. cordata’s* niche specialization in hydromorphic soils. Ecophysiological studies demonstrate that the species’ characteristic distribution in flood-prone, organic-rich substrates (Shi, 2014) creates optimal conditions for volatile compound synthesis through anaerobic soil microbe interactions and redox-sensitive enzymatic processes. Slope-mediated drainage patterns further regulate soil aeration and nutrient mobility, creating microhabitat gradients that directly influence terpenoid precursor availability. These findings highlight the critical role of pedological conditions in maintaining the biochemical integrity of medicinal plants, particularly for lipophilic compounds like 2-Undecanone that exhibit soil matrix-dependent stabilization.

### 4.3 Conservation strategies for *H. cordata*

The medicinal significance of *H. cordata* has been amplified in contemporary therapeutics, particularly through its demonstrated antiviral efficacy in COVID-19 management, which bridges traditional phytomedicine and modern pharmacotherapy. However, anthropogenic habitat fragmentation and climate-driven biogeographic shifts have precipitated a transition from contiguous populations to disjunct metapopulations, threatening the species’ ecological resilience. This study advances a conservation framework integrating habitat suitability modeling (HSI), spatial metabolite profiling (SQIs), and ecological quality indices (EQIs) to reconcile sustainable utilization with biodiversity preservation. The proposed zoning strategy identifies southwestern Hubei as a novel priority area for cultivation, supplementing established Good Agricultural Practice (GAP) bases, while revealing metabolite-specific biogeochemical optima: traditional production zones like Sichuan and Guizhou maximize 2-Undecanone yields, whereas northern ecoregions such as Hubei favor quercetin biosynthesis.

Such geolocation-driven cultivation protocols enable tiered resource allocation—plants from high-metabolite geographies supply precision pharmaceutical applications, while pharmacopeia-compliant low-yield specimens serve general herbal markets. This stratification optimizes commercial viability without compromising therapeutic standards. Critically, SMs optimization requires multivariate agroecological management, as biosynthetic pathways respond nonlinearly to cultivation chronosequences, microclimate modulation, and edaphic engineering. Implementation necessitates rigorous ecotype evaluation to prevent invasive displacement of native flora, ensuring cultivation landscapes mirror natural community assemblages. By calibrating production landscapes to both metabolic gradients and regional carrying capacity, this paradigm shift in medicinal plant management balances anthropogenic demand with biome conservation, establishing a replicable model for climate-resilient phytopharmacology.

### 4.4 Future outlook

This study underscores the critical role of identifying key environmental drivers in shaping both the ecological niche dynamics and phytochemical quality of *H. cordata*. A precise delineation of these variables enhances the predictive capacity of ecological niche models while elucidating their regulatory mechanisms on the biosynthesis of quality-defining SMs.

While centered on a medicinal species, the “Ecological Suitability - Spatial Quality Zoning” framework demonstrates transferability to agronomically vital crops - including tomato, potato, and rice - whose productivity and phenotypic plasticity exhibit strong environmental dependencies tied to thermal regimes, hydrological cycles, and pedological characteristics (Mahmood et al., 2012.; Rykaczewska, 2013; Timlin et al., 2006; van der Ploeg and Heuvelink, 2005). By synergistically evaluating ecological constraints and metabolite gradients, this dual-axis framework provides actionable insights for precision agriculture, enabling spatially optimized crop zoning, cultivation protocols, and resource allocation strategies that concurrently support yield maximization and ecosystem integrity.

However, traditional SDMs operate under the assumption that a species exhibits homogeneous environmental requirements—an assumption not met in many medicinal plants. The sample data in this study, for instance, did not account for interregional germplasm variation. In reality, many nominal “species” encompass multiple chemotypes or ecotypes that have undergone genetic differentiation, resulting in distinct environmental demands and response patterns.

The formation of “genuine regional herbs” exemplifies this complexity: it arises from Genotype (G) × Environment (E) interactions, where specific genetic germplasms adapt to and interact with unique local conditions to produce high-quality medicinal materials. Incorporating germplasm data into ecological niche modeling could therefore significantly improve predictive accuracy. Such integration would allow not only the identification of spatially suitable habitats but also the projection of zones conducive to high accumulation of target bioactive compounds—enabling a more scientifically-grounded approach to the conservation and sustainable utilization of genuine regional herbs resources.

## 5 Conclusion

This study investigates the ecological suitability and phytochemical quality distribution patterns of *H. cordata* by integrating species distribution modeling with SMs spatial analysis.

(1) Employing occurrence records and environmental variables, Biomod2 ensemble modeling identified optimal growth habitats, revealing high-suitability zones concentrated in subtropical monsoon regions of eastern Sichuan (Ya’an), Chongqing-Hubei borderlands, and southern Yunnan, with Bio12 and Bio17 emerging as primary bioclimatic determinants.
(2) Path analysis coupled with Kriging interpolation elucidated SMs-environment relationships, demonstrating distinct spatial heterogeneity in three key metabolites: 2-Undecanone concentrations correlated strongly with Bio7, Sq4, and slope gradients; quercitrin and quercetin distributions exhibited Bio7 associations with Sand2 and Den.
(3) The EQI, synthesized from habitat suitability and SMs spatial patterns, delineated metabolite-specific cultivation hotspots - 2-Undecanone-rich areas clustered in Guizhou-Yunnan-Fujian transitional zones, quercitrin-abundant regions spanning southwestern to eastern provinces, and quercetin-concentrated pockets in northern peripheries of the species’ range. Notably, SM optimization zones displayed minimal geographical overlap, underscoring the necessity for cultivation strategies tailored to target compounds.

These findings advance precision agriculture paradigms by establishing an ecological-quality dual assessment framework applicable to medicinal plants and economically vital crops, while highlighting microclimatic and edaphic factors requiring prioritized consideration in conservation-oriented cultivation practices.

## Data Availability

The original contributions presented in the study are included in the article/**Supplementary Material**. Further inquiries can be directed to the corresponding author.
