# Supplementary material for: Using ecological niche modelling to identify the high-quality ecological areas of Houttuynia cordata Thunb. based on secondary metabolites content
Source: Front Plant Sci. 2025 Oct 14;16:1641634. doi: 10.3389/fpls.2025.1641634 (PMC12558978; doi:10.3389/fpls.2025.1641634)
Supplement: Supplementary file 1 [file Table1.docx]

Appendix

**Table S1** Samples of *H.cordata*

| Specials | Longtitude | Latitude |
| --- | --- | --- |
| *H.cordata* | 101.56752 | 21.475834 |
| *H.cordata* | 107.325268 | 22.010392 |
| *H.cordata* | 109.876746 | 22.082121 |
| *H.cordata* | 107.957979 | 22.097768 |
| *H.cordata* | 110.264143 | 22.238806 |
| *H.cordata* | 111.031652 | 22.446542 |
| *H.cordata* | 107.839351 | 22.464142 |
| *H.cordata* | 101.049998 | 22.504017 |
| *H.cordata* | 110.165470 | 22.638462 |
| *H.cordata* | 113.938254 | 22.696199 |
| *H.cordata* | 100.860736 | 22.697654 |
| *H.cordata* | 109.207192 | 22.755060 |
| *H.cordata* | 103.086573 | 22.792186 |
| *H.cordata* | 110.621080 | 22.845607 |
| *H.cordata* | 112.044025 | 22.929376 |
| *H.cordata* | 103.687613 | 22.983563 |
| *H.cordata* | 104.394161 | 23.012914 |
| *H.cordata* | 113.341527 | 23.127041 |
| *H.cordata* | 104.644532 | 23.180546 |
| *H.cordata* | 102.835218 | 23.219942 |
| *H.cordata* | 111.266023 | 23.250130 |
| *H.cordata* | 111.900000 | 23.280000 |
| *H.cordata* | 107.580539 | 23.327903 |
| *H.cordata* | 104.250727 | 23.363000 |
| *H.cordata* | 114.269636 | 23.376591 |
| *H.cordata* | 104.672252 | 23.437468 |
| *H.cordata* | 110.462487 | 23.519195 |
| *H.cordata* | 105.630906 | 23.625319 |
| *H.cordata* | 102.826592 | 23.634745 |
| *H.cordata* | 108.232005 | 23.636406 |
| *H.cordata* | 113.671053 | 23.651079 |
| *H.cordata* | 113.045465 | 23.688233 |
| *H.cordata* | 109.213028 | 23.695857 |
| *H.cordata* | 114.699884 | 23.743260 |
| *H.cordata* | 115.701646 | 23.781005 |
| *H.cordata* | 100.092756 | 23.880344 |
| *H.cordata* | 107.410192 | 23.960200 |
| *H.cordata* | 110.991129 | 24.095943 |
| *H.cordata* | 117.148786 | 24.138290 |
| *H.cordata* | 110.235565 | 24.175587 |
| *H.cordata* | 113.415062 | 24.186045 |
| *H.cordata* | 102.239789 | 24.271311 |
| *H.cordata* | 102.970004 | 24.276807 |
| *H.cordata* | 109.343000 | 24.303000 |
| *H.cordata* | 116.625881 | 24.33566 |
| *H.cordata* | 116.346712 | 24.341581 |
| *H.cordata* | 114.488708 | 24.369586 |
| *H.cordata* | 106.006265 | 24.388378 |
| *H.cordata* | 106.633387 | 24.392902 |
| *H.cordata* | 113.987821 | 24.399048 |
| *H.cordata* | 111.54332 | 24.428309 |
| *H.cordata* | 114.938686 | 24.442178 |
| *H.cordata* | 107.410176 | 24.518994 |
| *H.cordata* | 115.57351 | 24.521543 |
| *H.cordata* | 112.080663 | 24.567694 |
| *H.cordata* | 99.928540 | 24.580446 |
| *H.cordata* | 100.709787 | 24.615701 |
| *H.cordata* | 98.644444 | 24.670000 |
| *H.cordata* | 108.085567 | 24.692597 |
| *H.cordata* | 117.310000 | 24.700000 |
| *H.cordata* | 102.853436 | 24.751500 |
| *H.cordata* | 112.894000 | 24.760100 |
| *H.cordata* | 110.451522 | 24.846283 |
| *H.cordata* | 115.650136 | 24.95476 |
| *H.cordata* | 109.172452 | 25.031795 |
| *H.cordata* | 109.926496 | 25.032990 |
| *H.cordata* | 104.890000 | 25.080000 |
| *H.cordata* | 105.390000 | 25.130000 |
| *H.cordata* | 115.393921 | 25.136922 |
| *H.cordata* | 116.890537 | 25.141368 |
| *H.cordata* | 98.848333 | 25.159722 |
| *H.cordata* | 110.351669 | 25.175621 |
| *H.cordata* | 114.330000 | 25.180000 |
| *H.cordata* | 105.610312 | 25.255682 |
| *H.cordata* | 107.681433 | 25.379048 |
| *H.cordata* | 117.830678 | 25.384913 |
| *H.cordata* | 118.892935 | 25.385889 |
| *H.cordata* | 106.447573 | 25.402958 |
| *H.cordata* | 99.541240 | 25.464684 |
| *H.cordata* | 111.600795 | 25.526439 |
| *H.cordata* | 106.766734 | 25.554789 |
| *H.cordata* | 100.090375 | 25.572598 |
| *H.cordata* | 115.786060 | 25.600277 |
| *H.cordata* | 110.585686 | 25.601680 |
| *H.cordata* | 114.765114 | 25.661237 |
| *H.cordata* | 108.435928 | 25.671026 |
| *H.cordata* | 107.712034 | 25.718529 |
| *H.cordata* | 105.116204 | 25.730505 |
| *H.cordata* | 110.040007 | 25.742153 |
| *H.cordata* | 116.357607 | 25.833607 |
| *H.cordata* | 118.484192 | 25.843033 |
| *H.cordata* | 118.801990 | 25.856800 |
| *H.cordata* | 104.845324 | 25.864129 |
| *H.cordata* | 111.009947 | 25.897942 |
| *H.cordata* | 113.144988 | 25.922374 |
| *H.cordata* | 114.200556 | 25.934892 |
| *H.cordata* | 117.359304 | 25.959029 |
| *H.cordata* | 108.450000 | 26.000000 |
| *H.cordata* | 108.128373 | 26.033258 |
| *H.cordata* | 107.171128 | 26.135443 |
| *H.cordata* | 112.879378 | 26.227622 |
| *H.cordata* | 105.141973 | 26.255937 |
| *H.cordata* | 114.514242 | 26.324960 |
| *H.cordata* | 115.360000 | 26.330000 |
| *H.cordata* | 117.651005 | 26.339452 |
| *H.cordata* | 108.038474 | 26.404449 |
| *H.cordata* | 106.740244 | 26.409179 |
| *H.cordata* | 110.850000 | 26.430000 |
| *H.cordata* | 111.316667 | 26.466667 |
| *H.cordata* | 107.592000 | 26.601000 |
| *H.cordata* | 114.26354 | 26.602915 |
| *H.cordata* | 118.102167 | 26.642077 |
| *H.cordata* | 110.630000 | 26.720000 |
| *H.cordata* | 106.745000 | 26.738000 |
| *H.cordata* | 116.325752 | 26.837264 |
| *H.cordata* | 108.483284 | 26.876969 |
| *H.cordata* | 117.170000 | 26.900000 |
| *H.cordata* | 109.511000 | 26.905000 |
| *H.cordata* | 118.190000 | 26.990000 |
| *H.cordata* | 107.903353 | 27.053521 |
| *H.cordata* | 110.575848 | 27.060320 |
| *H.cordata* | 106.731000 | 27.068000 |
| *H.cordata* | 107.262304 | 27.071219 |
| *H.cordata* | 109.229782 | 27.112233 |
| *H.cordata* | 116.520000 | 27.210000 |
| *H.cordata* | 111.400000 | 27.224000 |
| *H.cordata* | 109.752000 | 27.233000 |
| *H.cordata* | 112.730711 | 27.241564 |
| *H.cordata* | 105.280000 | 27.300000 |
| *H.cordata* | 108.845946 | 27.322705 |
| *H.cordata* | 118.120000 | 27.330000 |
| *H.cordata* | 117.492653 | 27.340665 |
| *H.cordata* | 108.055954 | 27.341579 |
| *H.cordata* | 105.554254 | 27.386259 |
| *H.cordata* | 114.610000 | 27.390000 |
| *H.cordata* | 115.500000 | 27.400000 |
| *H.cordata* | 117.798000 | 27.415000 |
| *H.cordata* | 104.873643 | 27.441640 |
| *H.cordata* | 113.814589 | 27.469256 |
| *H.cordata* | 110.083000 | 27.489000 |
| *H.cordata* | 116.222122 | 27.546148 |
| *H.cordata* | 119.659000 | 27.551000 |
| *H.cordata* | 119.930000 | 27.560000 |
| *H.cordata* | 108.850000 | 27.690000 |
| *H.cordata* | 107.198169 | 27.783956 |
| *H.cordata* | 110.084000 | 27.787000 |
| *H.cordata* | 120.470000 | 27.820000 |
| *H.cordata* | 106.563194 | 27.859770 |
| *H.cordata* | 116.360064 | 27.977282 |
| *H.cordata* | 106.920836 | 28.050895 |
| *H.cordata* | 108.686504 | 28.084769 |
| *H.cordata* | 105.164749 | 28.088164 |
| *H.cordata* | 119.409000 | 28.102000 |
| *H.cordata* | 112.676159 | 28.133153 |
| *H.cordata* | 109.200000 | 28.150000 |
| *H.cordata* | 112.001598 | 28.176082 |
| *H.cordata* | 111.998504 | 28.178101 |
| *H.cordata* | 119.839000 | 28.206000 |
| *H.cordata* | 113.228971 | 28.286507 |
| *H.cordata* | 117.245492 | 28.292516 |
| *H.cordata* | 117.709450 | 28.315215 |
| *H.cordata* | 107.822569 | 28.316457 |
| *H.cordata* | 114.780982 | 28.385228 |
| *H.cordata* | 107.267701 | 28.401269 |
| *H.cordata* | 119.597000 | 28.417000 |
| *H.cordata* | 113.451041 | 28.482089 |
| *H.cordata* | 118.823219 | 28.509686 |
| *H.cordata* | 114.370980 | 28.520750 |
| *H.cordata* | 108.737000 | 28.521000 |
| *H.cordata* | 108.071899 | 28.647960 |
| *H.cordata* | 104.010884 | 28.672732 |
| *H.cordata* | 119.866000 | 28.678000 |
| *H.cordata* | 115.380000 | 28.700000 |
| *H.cordata* | 104.559000 | 28.770000 |
| *H.cordata* | 113.083595 | 28.815762 |
| *H.cordata* | 107.583333 | 28.816667 |
| *H.cordata* | 110.451596 | 28.851070 |
| *H.cordata* | 117.977601 | 28.862278 |
| *H.cordata* | 105.385000 | 28.880000 |
| *H.cordata* | 108.658379 | 28.890891 |
| *H.cordata* | 114.999554 | 28.930219 |
| *H.cordata* | 117.281101 | 28.981283 |
| *H.cordata* | 109.850000 | 29.000000 |
| *H.cordata* | 114.412629 | 29.010101 |
| *H.cordata* | 107.165668 | 29.019444 |
| *H.cordata* | 119.63238 | 29.031361 |
| *H.cordata* | 115.822033 | 29.124451 |
| *H.cordata* | 118.410000 | 29.130000 |
| *H.cordata* | 103.909000 | 29.153000 |
| *H.cordata* | 109.246614 | 29.200862 |
| *H.cordata* | 108.193872 | 29.245053 |
| *H.cordata* | 117.862004 | 29.247988 |
| *H.cordata* | 106.266667 | 29.300000 |
| *H.cordata* | 104.699000 | 29.348000 |
| *H.cordata* | 108.687476 | 29.365795 |
| *H.cordata* | 120.822222 | 29.390841 |
| *H.cordata* | 110.694641 | 29.391403 |
| *H.cordata* | 111.130000 | 29.420000 |
| *H.cordata* | 114.200000 | 29.433333 |
| *H.cordata* | 119.330000 | 29.480000 |
| *H.cordata* | 103.398589 | 29.496119 |
| *H.cordata* | 117.373176 | 29.534772 |
| *H.cordata* | 118.870000 | 29.570000 |
| *H.cordata* | 120.506000 | 29.576000 |
| *H.cordata* | 103.688000 | 29.579000 |
| *H.cordata* | 104.993000 | 29.583000 |
| *H.cordata* | 121.410000 | 29.590000 |
| *H.cordata* | 115.66106 | 29.597189 |
| *H.cordata* | 109.150000 | 29.670000 |
| *H.cordata* | 110.736604 | 29.697564 |
| *H.cordata* | 118.245000 | 29.710000 |
| *H.cordata* | 102.846741 | 29.792928 |
| *H.cordata* | 110.100000 | 29.833333 |
| *H.cordata* | 104.121875 | 29.852190 |
| *H.cordata* | 114.291554 | 29.857250 |
| *H.cordata* | 121.540000 | 29.860000 |
| *H.cordata* | 106.353302 | 29.865426 |
| *H.cordata* | 103.098000 | 29.943000 |
| *H.cordata* | 109.480000 | 29.990000 |
| *H.cordata* | 120.594000 | 30.000000 |
| *H.cordata* | 107.795470 | 30.006410 |
| *H.cordata* | 109.016667 | 30.016667 |
| *H.cordata* | 108.185012 | 30.023401 |
| *H.cordata* | 104.602000 | 30.125000 |
| *H.cordata* | 118.502000 | 30.145000 |
| *H.cordata* | 119.651811 | 30.168553 |
| *H.cordata* | 117.301000 | 30.178000 |
| *H.cordata* | 119.077000 | 30.188000 |
| *H.cordata* | 109.204000 | 30.281000 |
| *H.cordata* | 103.134000 | 30.379000 |
| *H.cordata* | 103.579205 | 30.431945 |
| *H.cordata* | 116.981000 | 30.536000 |
| *H.cordata* | 114.159956 | 30.568041 |
| *H.cordata* | 117.927793 | 30.622513 |
| *H.cordata* | 103.935000 | 30.658000 |
| *H.cordata* | 111.923000 | 30.697000 |
| *H.cordata* | 111.156569 | 30.704411 |
| *H.cordata* | 106.459000 | 30.759000 |
| *H.cordata* | 116.500000 | 30.760000 |
| *H.cordata* | 106.082997 | 30.791258 |
| *H.cordata* | 109.751000 | 30.810000 |
| *H.cordata* | 121.433857 | 30.847479 |
| *H.cordata* | 119.073000 | 30.896000 |
| *H.cordata* | 103.549637 | 30.907117 |
| *H.cordata* | 110.381000 | 30.908000 |
| *H.cordata* | 117.752000 | 30.937000 |
| *H.cordata* | 108.831983 | 30.954306 |
| *H.cordata* | 109.410000 | 31.050000 |
| *H.cordata* | 121.695706 | 31.104830 |
| *H.cordata* | 109.879450 | 31.114073 |
| *H.cordata* | 111.291000 | 31.128000 |
| *H.cordata* | 120.406916 | 31.237023 |
| *H.cordata* | 108.136000 | 31.256000 |
| *H.cordata* | 115.936000 | 31.275000 |
| *H.cordata* | 103.947992 | 31.296233 |
| *H.cordata* | 118.253000 | 31.328000 |
| *H.cordata* | 110.780000 | 31.330000 |
| *H.cordata* | 121.243798 | 31.360105 |
| *H.cordata* | 119.820000 | 31.370000 |
| *H.cordata* | 109.629918 | 31.370137 |
| *H.cordata* | 119.380789 | 31.420469 |
| *H.cordata* | 104.582000 | 31.467000 |
| *H.cordata* | 120.299456 | 31.579934 |
| *H.cordata* | 111.502000 | 31.619000 |
| *H.cordata* | 120.833688 | 31.662505 |
| *H.cordata* | 113.554000 | 31.736000 |
| *H.cordata* | 115.400000 | 31.790000 |
| *H.cordata* | 110.648810 | 31.790636 |
| *H.cordata* | 108.629497 | 31.824099 |
| *H.cordata* | 106.685000 | 31.857000 |
| *H.cordata* | 118.567479 | 32.047270 |
| *H.cordata* | 108.040000 | 32.060000 |
| *H.cordata* | 114.080000 | 32.100000 |
| *H.cordata* | 119.212993 | 32.123908 |
| *H.cordata* | 110.712211 | 32.144677 |
| *H.cordata* | 111.190000 | 32.600000 |
| *H.cordata* | 115.991927 | 23.917350 |
| *H.cordata* | 110.153599 | 29.395292 |
